# Case Report: A Rare Case of Elderly-Onset Adult-Onset Still’s Disease in a Patient With Systemic Lupus Erythematosus

**DOI:** 10.3389/fimmu.2022.822169

**Published:** 2022-01-18

**Authors:** Yasuaki Hirooka, Saki Okuda, Masafumi Sugiyama, Toshihiko Shiga, Yuji Nozaki, Koji Kinoshita, Masanori Funauchi, Itaru Matsumura

**Affiliations:** ^1^ Department of Rheumatology, Kindai University Nara Hospital, Nara, Japan; ^2^ Department of Hematology and Rheumatology, Kindai University School of Medicine, Osaka, Japan

**Keywords:** adult-onset Still’s disease, rheumatic diseases, systemic lupus erythematosus, overlap, case report

## Abstract

The rare systemic inflammatory disorder ‘adult-onset Still’s disease (AOSD)’ is characterized by recurrent fever, evanescent rash, arthralgia, and leukocytosis with neutrophilia. The Yamaguchi criteria are widely used to diagnose AOSD; these criteria can be used for diagnosis after a wide range of infectious, rheumatic, and neoplastic diseases have been excluded. AOSD generally does not overlap with other rheumatic diseases. We present the rare case of an 80-year-old Japanese woman who presented with arthralgia, fever, and skin rash during treatment for systemic lupus erythematosus (SLE), which was finally diagnosed as an overlap of AOSD. Blood tests revealed leukocytosis with neutrophilia, high C-reactive protein (CRP), and liver dysfunction. Her anti-ds-DNA antibody titer and serum complement titer were at the same level as before and remained stable. We suspected AOSD based on the high serum ferritin level but hesitated to diagnose AOSD because of the patient’s SLE history. We measured serum interleukin (IL)-18; it was extremely high at 161,221 pg/mL, which was strongly suggestive of AOSD. We thus diagnosed AOSD complicated during the course of treatment for SLE. The patient’s arthralgia and high CRP level persisted after we increased her oral prednisolone dose and added oral methotrexate, but her symptoms eventually improved with the addition of intravenous tocilizumab. We note that the presence of autoantibodies or other rheumatic diseases cannot be absolutely ruled out in the diagnosis of AOSD. Although high serum IL-18 levels are not specific for AOSD, the measurement of serum IL-18 may aid in the diagnosis of AOSD in similar rare cases.

## Introduction

Adult-onset Still’s disease (AOSD) is a rare systemic inflammatory disorder characterized by recurrent fever, evanescent rash, arthralgia or arthritis, sore throat, leukocytosis with neutrophilia, lymphadenopathy, splenomegaly, and liver dysfunction (1). AOSD is often present in young adults, with a prevalence of 1 to 34 per million people in Japan and Europe (1). The etiology of AOSD is unknown, but abnormal activation of innate immune cells (e.g., neutrophils, monocytes, and macrophages) and the excessive productions of cytokines such as interleukin (IL)-1β, IL-6, tumor necrosis factor-alpha (TNF-α), and IL-18 are thought to play important roles (1).

The Yamaguchi criteria, proposed in 1992, have a sensitivity of 96% and specificity of 92% and are widely used for the diagnosis of AOSD (2). AOSD is a diagnosis of exclusion, and the criteria can be used for the diagnosis of AOSD after a wide range of infectious, rheumatic, and neoplastic diseases have been excluded. There are four major Yamaguchi criteria: (*i*) fever ≥39°C lasting ≥1 week, (*ii*) arthralgia or arthritis lasting ≥2 weeks, (*iii*) typical nonpruritic salmon-colored rash, and (*iv*) leukocytosis ≥10,000/mm, with granulocytes ≥80%. There are four minor Yamaguchi criteria: (*i*) sore throat, (*ii*) lymphadenopathy and/or splenomegaly, (*iii*) abnormal liver function test results, and (*iv*) negative test results for antinuclear antibody and rheumatoid factor.

To be diagnosed with AOSD, a patient must meet five of these criteria, including at least two of the major criteria. Because there are no specific tests that can clearly distinguish AOSD from similar disorders, diagnosing AOSD may be difficult if the patient’s symptoms are not typical. Other rheumatic diseases are excluded by Yamaguchi’s criteria, and AOSD generally does not overlap with them. We present here a rare case of arthralgia, fever, and skin rash during the course of treatment for systemic lupus erythematosus (SLE), which was finally diagnosed as an overlap of AOSD.

## Case Presentation

An 80-year-old Japanese woman who regularly attended our hospital for SLE visited our hospital because of fatigue and arthralgia. She had been referred to our hospital at the age of 77 with hemolytic anemia and was diagnosed with SLE based on the results of tests including leukopenia, positive direct Coombs test, positive antinuclear antibody, positive anti-dsDNA antibody, and hypocomplementemia. As treatment for SLE, she received oral prednisolone (PSL) 30 mg (0.6 mg/kg), which improved her hemolytic anemia, and the PSL dose was later reduced.

When the patient came to our hospital for fatigue and arthralgia, she was taking 5 mg/day of oral PSL. The blood tests administered at that time showed an elevated white blood cell (WBC) count of 11,600/μL, neutrophils at 83.3%, and C-reactive protein (CRP) at 6.5 mg/dL. She was given 1,000 mg/day of oral cephalexin for a suspected bacterial infection. Seven days later, she underwent blood tests again and the data did not improve: WBC 14,900 μL, neutrophils 91.6%, and CRP 8.0 mg/dL. Her arthralgia was very severe in her hands and knees, making it difficult for her to perform her daily activities. She was thus admitted to our hospital.

The results of the laboratory investigation on admission (Day 1) are shown in **Table 1**. The blood tests showed leukocytosis with neutrophilia, high CRP, and mild liver dysfunction. Two sets of blood cultures were taken for the possible bacterial infection, and the patient was started on intravenous piperacillin/tazobactam 9 g/day. On the night of Day 1, she had a fever of 39.4°C, and continued to have a fever of 38°C or higher the next day. Computed tomography scans of the neck, chest, abdomen, and pelvis taken on admission showed no obvious abnormalities. Echocardiography showed no warts suggestive of infective endocarditis.

**Table 1:** Laboratory data on admission.

	Result	Reference interval
**Complete blood counts**		
White blood cells	14900/μL	3300-8600
Nuetrophils	91.6%	38-77
Lymphocytes	3.3%	20.2-53.2
Monocytes	3.6%	2.7-9.3
Eosinophils	1.3%	0.2-4.1
Basophils	0.1%	0.2-1.3
Red blood cells	404×10^4^ /μL	435×10^4^ -555×10^4^
Hemoglobin	12.3 g/dL	13.7-16.8
Hematocrit	37.6%	40.7-50.1
Platelets	29.1×10^4^ /μL	15.8-34.8
**Urinalysis**		
Protein	–	–
Occult blood	–	–
**Biochemistry**		
CRP	8.03 mg/dL	0-0.14
Blood urea nitrogen	27.6 mg/dL	8.0-20.0
Creatinine	0.86 mg/dL	0.65-1.07
Total protein	6.8 g/dL	6.6-8.1
Albumin	3.5 g/dL	4.1-5.1
Total bilirubin	0.4 mg/dL	0.4-1.5
γ-glutamyltransferase	31 U/L	13-64
Aspartate transaminase	45 U/L	13-30
Alanine aminotransferase	15 U/L	10-42
Lactate dehydrogenase	539 U/L	124-222
Creatinine kinase	52 U/L	59-248
Ferritin	11454 ng/mL	5.0~157.0
**Immunology**		
Rheumatoid factor	4 IU/mL	0-15
anti-cyclic citrullinated peptide antibody	0.8 U/mL	0-4.4
Anti-ds-DNA antibody	35.3 IU/mL	0-9.9
C3	142 mg/dL	80-140
C4	25.9 mg/dL	11-34
CH50	49 U/mL	30-45
IgG	1273 mg/dL	861-1747
IL-6	115 pg/mL	0-7
**Tumor markers**		
CEA	2.5 ng/mL	0-5
CA19-9	9.5 U/mL	0-37
Soluble IL-2 receptor	986 U/mL	122-496

**Figure 1:**
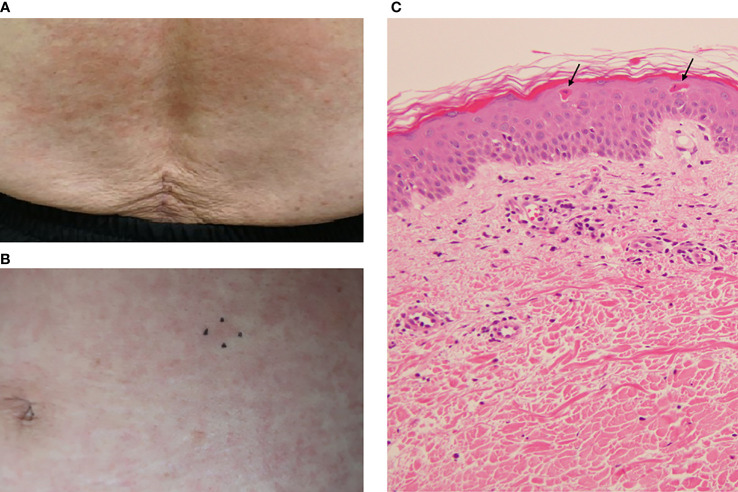
The patient’s skin rash and its pathological findings. **(A)** Skin rash on the lower back. **(B)** Skin rash on the abdomen. The mark indicates the site of the skin biopsy. **(C)** Pathological findings of the abdominal skin. Hematoxylin and eosin (H&E) staining. Original magnification: ×200. *Arrows* indicate dyskeratotic cells in the upper epidermis.

The day after her admission (Day 2), the patient developed a pruritic, persistent, erythematous skin rash on her anterior chest, abdomen, and back (**Figures 1A, B**). On Day 3, the skin rash spread to both upper limbs and both thighs, and redness and edema appeared on both eyelids. The skin rash on the patient’s back was also accompanied by linear erythema that could be scratching scars. On Day 5, since the patient’s fever persisted, the antibiotics were changed to meropenem 2 g/day and daptomycin 300 mg/day as the piperacillin/tazobactam was considered ineffective. Diffusion-weighted whole-body imaging with background body signal suppression (DWIBS) was performed on the same day, but there were no findings suggestive of malignancy, inflammation, or abscess. On the same day, the serum ferritin level was high at 11,454 ng/mL.

For the examination of potential malignant lymphoma and hemophagocytic syndrome (HPS), we conducted a skin biopsy on Day 6 and a bone marrow biopsy on Day 8. The histopathological examination of the skin showed mild lymphocyte-dominated inflammatory cell infiltration around capillaries in the upper dermis and scattered dyskeratotic cells in the upper epidermis (**Figure 1C**). There were no findings of vasculitis or malignant lymphoma in the skin histopathology. The bone marrow biopsy showed no findings suggestive of malignant diseases or HPS. Blood cultures were persistently negative, and the patient’s fever was not brought down by antibiotics. Infectious disease was thus ruled out, and the meropenem and daptomycin were discontinued on Day 8.

The patient’s anti-dsDNA antibody titer measured at the time of her admission was not elevated compared to the past values, and her serum complement level was at the reference value; these results did not suggest worsening of the patient’s SLE. However, since there was no clear cause for her fever, skin rash, and arthralgia, we suspected that these symptoms may have been caused by the SLE, and we increased the PSL dose from 5 mg/day to 30 mg/day on Day 8. The fever resolved on Day 9. Although the patient’s arthralgia improved slightly, it was still severe, and on Day 12 her CRP level was still high at 7.0 mg/dL. Because her arthralgia was not improving sufficiently, on Day 16 we added oral methotrexate (MTX) 6 mg/week. For a further malignancy search, an upper gastrointestinal endoscopy was performed on Day 19 and a colonoscopy was performed on Day 20; both showed no abnormalities.

The patient’s high-grade fever, arthralgia, skin rash, leukocytosis with neutrophilia, and liver dysfunction and above-described findings in addition to her high serum interleukin (IL)-6 and serum ferritin levels made us suspect AOSD. However, Yamaguchi’s criteria include negative antinuclear antibodies, and rheumatic diseases are listed as exclusions. We were thus hesitant to diagnose AOSD in light of the patient’s SLE. On Day 23, the IL-18 level of the serum collected on Day 8 was observed to be extremely high at 161,221 pg/mL, which was strongly suggestive of AOSD. We therefore diagnosed AOSD complicated during the course of treatment for SLE.

We then administered tocilizumab (TCZ) 8 mg/kg intravenously for the patient’s refractory AOSD, on Day 26 after admission. After the TCZ administration, her arthralgia decreased quickly, and she was able to perform daily activities with ease by the Day 30. Her oral PSL was reduced to 25 mg/day on Day 31. Her arthralgia continued to improve gradually and had almost disappeared on Day 37. On Day 40, which was 14 days after the first injection of TCZ, the patient was scheduled for a second injection of TCZ, but it was postponed because her platelet count was low at 8.7×10^4^/μL. On Day 44, her blood cytomegalovirus antigen C7-HRP was positive in 27/87,900 cells, and she was given oral valganciclovir 450 mg/day.

**Figure 2:**
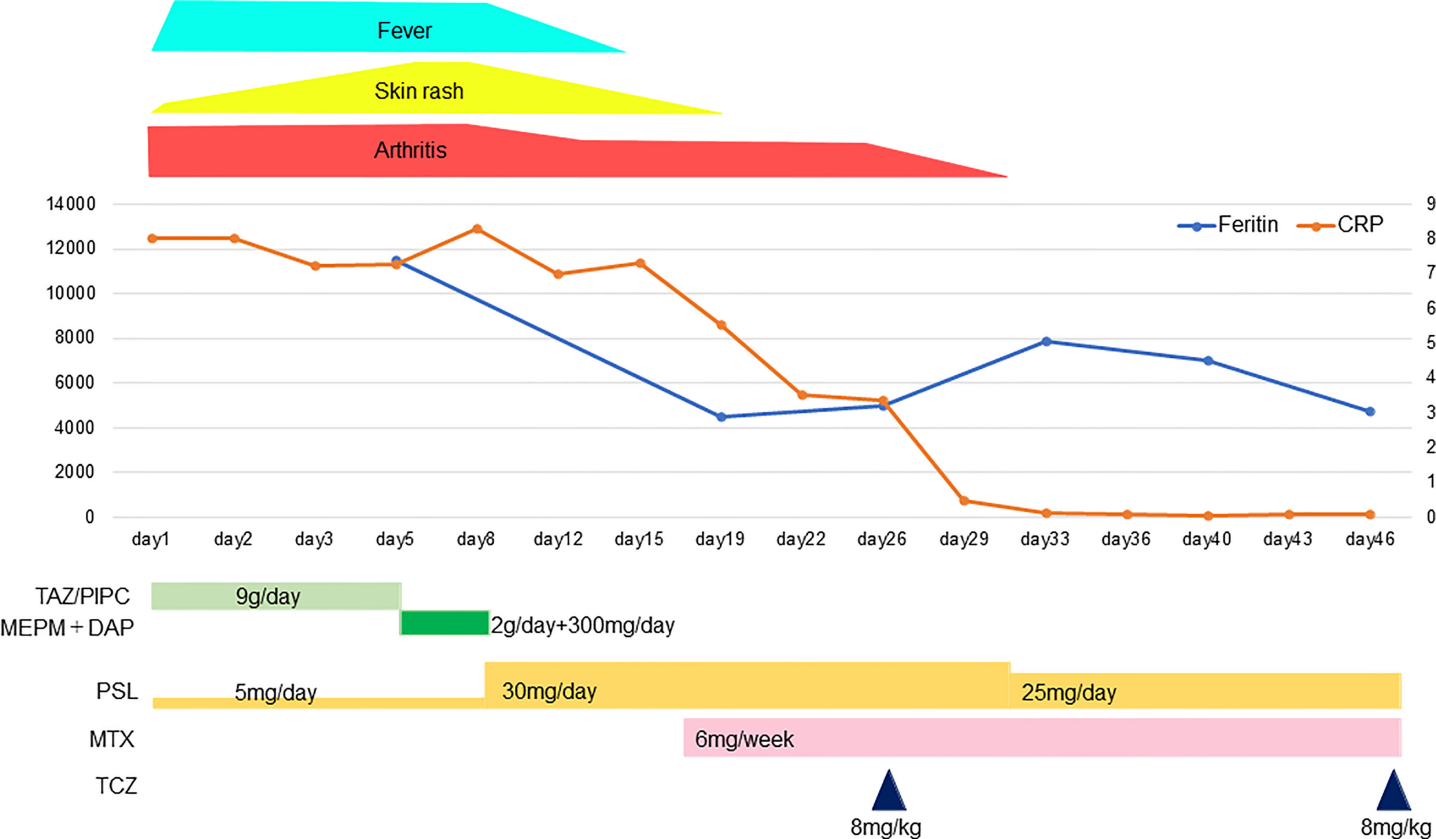
The patient’s clinical course. TAZ/PIPC, tazobactam/piperacillin; MEPM, meropenem; DAP, daptomycin; PSL, prednisolone; MTX, methotrexate; TCZ, tocilizumab.

After the valganciclovir was started, the patient’s platelet count recovered, and the cause was thought to be a cytomegalovirus infection rather than drug-induced by TCZ. On Day 47, a second dose of TCZ was administered and the oral PSL was reduced to 20 mg/day. On the 48th day after admission, The patient was discharged on Day 48; her clinical course is illustrated in **Figure 2**.

## Discussion

The Yamaguchi criteria are widely used for the diagnosis of AOSD. When these criteria are used, it is necessary to exclude other rheumatic diseases; AOSD thus generally does not overlap with other rheumatic diseases. Not much is known about AOSD as a comorbidity with autoimmune diseases, including SLE. To the best of our knowledge, the present case is the first case of AOSD overlapping with SLE.

As in the Yamaguchi criteria, autoantibodies are generally negative in AOSD. However, there have been several reports of autoantibody-positive AOSD cases (3–5). Zeng et al. retrospectively analyzed 61 Chinese patients with AOSD and reported that ANA was positive in seven of the 61 patients (11.5%) (6). Other immunological abnormalities described in patients with AOSD include rheumatoid factor, anti-cyclic citrullinated peptide antibody, anti-SS-A/Ro antibodies, anti-dsDNA antibodies, and anti-neutrophil cytoplasmic antibodies (4, 7–10). Although rare, there have been reports of an overlap of AOSD with other rheumatic diseases, such as Sjogren’s syndrome and systemic scleroderma (11–16). Although there have been reports of positive anti-dsDNA antibodies in patients with AOSD (8), there are no reports of AOSD cases with an overlap with SLE.

Our patient developed SLE at the age of 77 prior to the onset of AOSD. Late-onset SLE that develops after the age of 50 has been increasingly reported, affecting 2-20% of all patients with SLE (17). The onset of late-onset SLE is insidious, and uncommon clinical manifestations may lead to delayed diagnosis (17–19). Late-onset SLE has been reported to have less frequent skin manifestations, arthritis, and nephritis than early onset SLE (17–19). In our patient, there were no symptoms at the onset of SLE, including skin manifestations or arthritis, and no laboratory abnormalities suggestive of nephritis. The incidence of Sjögren’s syndrome has been reported to be higher in late-onset SLE than in early-onset SLE (19), but there are no reports of overlap with AOSD.

We suspected AOSD in our patient’s case because of the high serum ferritin level in addition to fever, arthralgia, skin rash, leukocytosis, and liver dysfunction. However, since the Yamaguchi criteria exclude SLE patients, there seemed to be insufficient evidence to diagnose this case as AOSD. While no definitive diagnosis could be made, the patient’s arthralgia remained severe even after the PSL dose was increased and MTX was added, and we were thus obliged to consider further intensified immunosuppressive therapy.

There are no disease-specific autoantibodies in AOSD to use for the diagnosis. The serum level of ferritin is frequently used as a practical tool for the diagnosis of AOSD, but its diagnostic specificity is low and of limited value in the diagnosis of AOSD (1). In our patient’s case, serum IL-18 was markedly elevated, which helped us reach the diagnosis of AOSD. IL-18 was found to be markedly elevated in the serum of AOSD patients in several studies (20, 21). Serum IL-18 was also elevated in patients with SLE, the median of which was significantly higher than that of healthy controls (265 vs. 169 pg/mL), but only at the 10³ level (22).

A subtype of hemophilic lymphocytosis (HLH) that complicates rheumatic diseases is called macrophage activation syndrome (MAS), and it is a life-threatening inflammatory disorder characterized by multiple organ failure, fever, and cytopenia, along with a marked elevation of serum IL-18 (23, 24). Complications of MAS during the course of SLE may increase the serum IL-18 level, but in our patient’s case, there was no cytopenia in the peripheral blood and no phagocytosis in the bone marrow as seen in MAS. Our patient did not meet the 2016 classification criteria for MAS in systemic juvenile idiopathic arthritis (sJIA) (25), and MAS was also excluded by the MAS/sJIA (MS) scoring system (26).

We reported that the serum IL-18 levels were significantly higher in patients with AOSD (with or without MAS) compared to patients with HLH secondary to other causes, including SLE (27), and we also observed that a serum IL-18 level >18,550 pg/mL could be used to distinguish between AOSD and HLH with 90.3% sensitivity and 93.5% specificity. Our patient’s remarkably high serum IL-18 level (161,221 pg/mL) was suggestive of AOSD. The Fautrel criteria proposed in 2002 included ferritin and glycosylated ferritin levels and do not require exclusion criteria as in the Yamaguchi criteria (28). Although glycosylated ferritin could not be measured in our hospital, our patient still met the Fautrel criteria. However, due to the unique presentation of this case of elderly onset SLE, we thought that the definitive diagnosis of this case as an overlap of AOSD should be made with great caution. We performed a variety of workups (including imaging and histological examinations) which adequately ruled out infection or malignancy. With the additional information provided by the patient’s high serum IL-18 level, we were finally able to reach the diagnosis of AOSD. It is important to note that the presence of autoantibodies or other rheumatic diseases cannot be completely ruled out in the diagnosis of AOSD.

AOSD usually affects young adults; the median age at diagnosis is approx. 36 years (1), but it can also occur in the elderly (29–31). The typical skin rash is an evanescent salmon-pink erythema, predominantly on the extremities, which is one of the diagnostic criteria. However, atypical skin rashes have been reported to be common in elderly-onset AOSD (29, 30). The most frequent lesions of atypical skin rashes are persistent papules, plaques, or erythema (30, 32, 33). This type of eruption is pruritic and has a linear configuration, probably due to the Koebner phenomenon, and it thus resembles flagellate erythema. Histopathologically, the eruption is characterized by the presence of singly or aggregated dyskeratotic/necrotic keratinocytes in the upper layers of the epidermis, accompanied by perivascular inflammatory infiltrates in the upper and middle dermis, without vasculitis (32, 33). SLE can present with various types of skin lesions, but unlike AOSD, in persistent pruritic eruptions of SLE, dyskeratotic keratinocytes are rarely found in the upper layers of the epidermis (34). The clinical and pathological findings of the rash in our patient’s case fit the atypical skin manifestations of AOSD. Other atypical skin rashes include urticaria, vesicular pustular eruptions, extensive peau d’orange appearance of the skin, and eyelid edema mimicking dermatomyositis (32, 33). Atypical skin rashes may delay the diagnosis and treatment of AOSD, and it should be noted that atypical skin rashes are more common in elderly patients.

Another characteristic of elderly-onset AOSD is that serum ferritin levels may be higher than in younger-onset AOSD. Maruyama et al. reported that serum ferritin levels were higher in elderly-onset AOSD patients without MAS or disseminated intravascular coagulation (DIC) (mean age at diagnosis: 71.2 years) than in younger-onset AOSD without MAS or DIC (mean age at diagnosis: 37.7 years), with median ferritin levels 7,943 ng/mL versus 2,492 ng/mL, respectively (29).

Glucocorticoids are the mainstay of treatment for AOSD, and disease-modifying anti-rheumatic drugs (DMARDS), including MTX, are added for disease control and glucocorticoid savings (1, 35). Biologics are considered for use in AOSD that is refractory to treatment with glucocorticoids and DMARDS. In Japan, tocilizumab, a humanized anti-IL-6 receptor antibody, is approved for the treatment of AOSD. Our patient’s arthralgia improved rapidly with the initiation of tocilizumab, and we were able to reduce the glucocorticoids.

There are few reports of AOSD overlapping with other rheumatic diseases. In the present case, oral MTX and intravenous tocilizumab were added because the patient’s arthralgia did not improve with only the increased dose of oral prednisolone. As of this writing, 4 months have passed since the patient’s AOSD onset, and she continues to receive these combination treatments without flare-up. The characteristics and long-term prognosis of AOSD in combination with other rheumatic diseases are not well understood. An accumulation of further cases is necessary.

Symptoms such as fever, arthralgia, and skin rash observed in the present case are common not only in AOSD but also in SLE patients. In our patient, neither the anti-dsDNA antibody titer nor the serum complement level suggested an exacerbation of SLE. However, SLE can flare-up despite unchanged serology. Therefore, we cannot be entirely excluded that this was an “AOSD-like” lupus flare, which is a limitation of our case report.

In conclusion, we have described a rare case of elderly-onset AOSD overlapping with SLE. We emphasize that the presence of autoantibodies or other rheumatic diseases is not completely excluded in the diagnosis of AOSD. Although high serum IL-18 levels are not specific for AOSD, the measurement of serum IL-18 may aid in the diagnosis of AOSD in similar rare cases.

## Data Availability Statement

The original contributions presented in the study are included in the article. Further inquiries can be directed to the corresponding author.

## Ethics Statement

Ethical review and approval was not required for the study on human participants in accordance with the local legislation and institutional requirements. The patients/participants provided their written informed consent to participate in this study. Written informed consent was obtained from the individual(s) for the publication of any potentially identifiable images or data included in this article.

## Author Contributions

YH, SO, and MS designed the study. YH and SO collected the data. YH drafted the manuscript. TS, YN, KK, MF, and IM edited the manuscript. All authors contributed to the manuscript’s revision and have read and approved the submitted version.

## Conflict of Interest

The authors declare that the research was conducted in the absence of any commercial or financial relationships that could be construed as a potential conflict of interest.

## Publisher’s Note

All claims expressed in this article are solely those of the authors and do not necessarily represent those of their affiliated organizations, or those of the publisher, the editors and the reviewers. Any product that may be evaluated in this article, or claim that may be made by its manufacturer, is not guaranteed or endorsed by the publisher.
