# Supplementary figures and images for: Case Report: A Rare Case of Elderly-Onset Adult-Onset Still’s Disease in a Patient With Systemic Lupus Erythematosus
Source: Front Immunol. 2022 Jan 18;13:822169. doi: 10.3389/fimmu.2022.822169 (PMC8803898; doi:10.3389/fimmu.2022.822169)

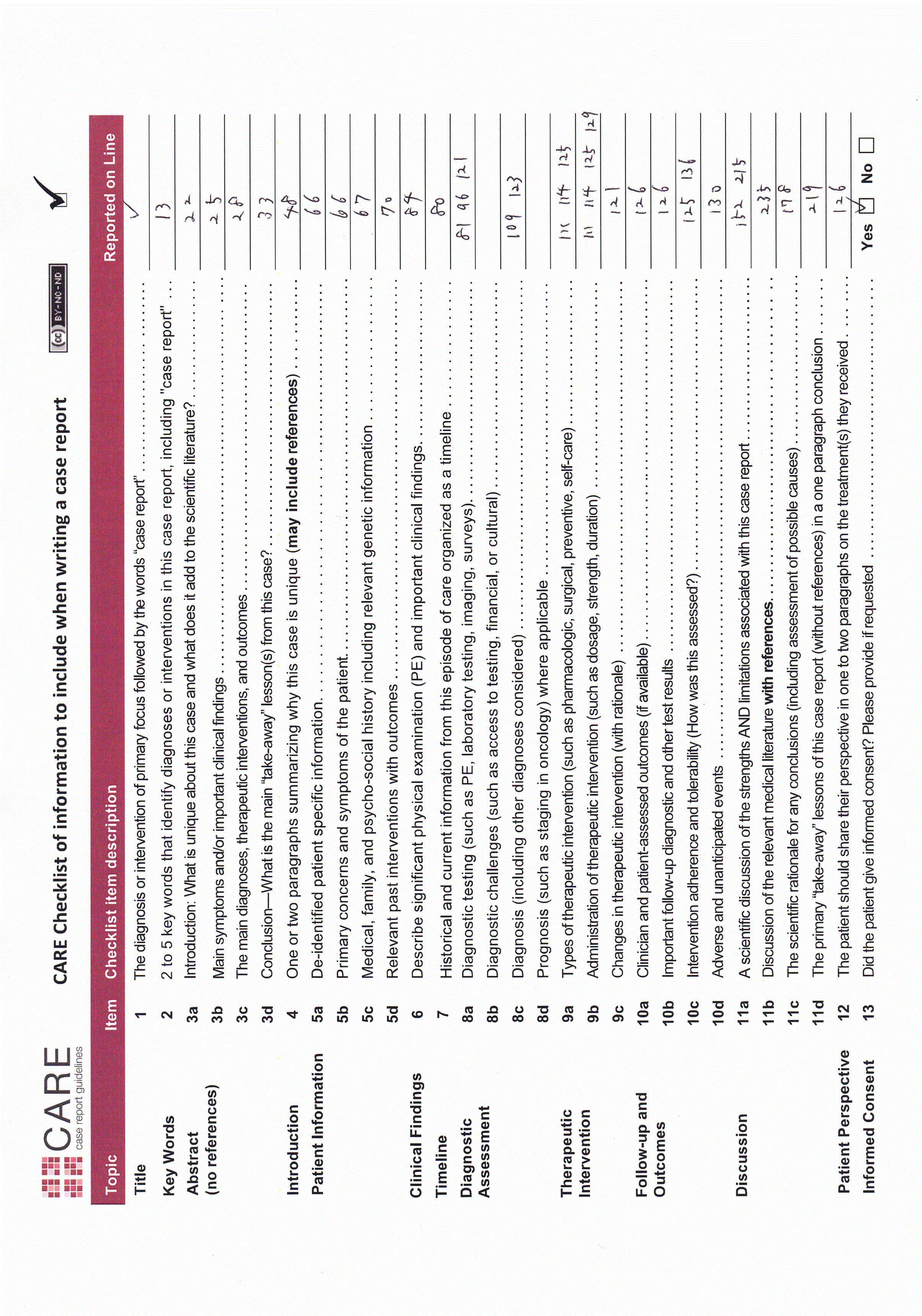

Supplement: Supplementary file 1 [file Image_1.tif]
